# The overexpression of DSP1 in neurons induces neuronal dysfunction and neurodegeneration phenotypes in *Drosophila*

**DOI:** 10.1186/s13041-024-01117-2

**Published:** 2024-07-13

**Authors:** Si-Eun Baek, Younghwi Kwon, Jong-Won Yoon, Hyo-Sung Kim, Jae-Yoon Yang, Dong-Seok Lee, Eunbyul Yeom

**Affiliations:** 1 School of Life Science and Biotechnology, College of Natural Sciences, Kyungpook National University, Daegu, 41566 South Korea; 2 School of Life Sciences, BK21 FOUR KNU Creative BioResearch Group, Kyungpook National University, Daegu, 41566 South Korea; 3 KNU-G LAMP Project Group, KNU Institute of Basic Sciences, School of Life Sciences, College of Natural Sciences, Kyungpook National University, Daegu, 41566 South Korea

**Keywords:** Neurodegeneration, DSP1, HMGB1, Neuromuscular junction, *Drosophila*

## Abstract

Dorsal switch protein 1(DSP1), a mammalian homolog of HMGB1, is firstly identified as a dorsal co-repressor in 1994. DSP1 contains HMG-box domain and functions as a transcriptional regulator in *Drosophila melanogaster*. It plays a crucial role in embryonic development, particularly in dorsal–ventral patterning during early embryogenesis, through the regulation of gene expression. Moreover, DSP1 is implicated in various cellular processes, including cell fate determination and tissue differentiation, which are essential for embryonic development. While the function of DSP1 in embryonic development has been relatively well-studied, its role in the adult *Drosophila* brain remains less understood. In this study, we investigated the role of DSP1 in the brain by using neuronal-specific DSP1 overexpression flies. We observed that climbing ability and life span are decreased in DSP1-overexpressed flies. Furthermore, these flies demonstrated neuromuscular junction (NMJ) defect, reduced eye size and a decrease in tyrosine hydroxylase (TH)-positive neurons, indicating neuronal toxicity induced by DSP1 overexpression. Our data suggest that DSP1 overexpression leads to neuronal dysfunction and toxicity, positioning DSP1 as a potential therapeutic target for neurodegenerative diseases.

**Supplementary Information:**

The online version contains supplementary material available at 10.1186/s13041-024-01117-2.

## Main text

Dorsal switch protein 1 (DSP1) was firstly identified in 1994 as a co-repressor of Dorsal protein in *Drosophila melanogaster* [1]. The DSP1 gene encodes a protein with a glutamine-rich domain (N-terminal), acidic tail (C-terminal) and two HMG (High Mobility Group) boxes designated as HMG box A and B [2]. HMG boxes are known to play a critical role in DNA binding including transcription factors and chromatin remodeling complexes [3]. DSP1 also has been shown to be involved in transcriptional activity and chromatin remodeling [1]. Previous studies have demonstrated that DSP1 binds to dorsal and affects transcriptional activity in dorsal. Dorsal is a key transcription factor responsible for dorso-ventral patterning during embryonic development in *Drosophila* [1, 4]. The activity of the Dorsal protein, which can act as both a transcriptional activator and repressor, varies depending on the promoter [4]. TATA-binding protein (TBP) is essential for transcription, being capable of direct interaction with the *Drosophila* transcription factor IIA (TFIIA). The TBP-TFIIA complex is involved in the regulation of various gene expressions, including Dorsal protein [5]. DSP1 directly binds to TBP, disrupting the TBP-TFIIA complex, particularly affecting TBP-gene interactions, which suppresses transcriptional activity of Dorsal protein [5]. In situ hybridization data indicated that DSP1 is expressed in the ovaries and brain of adult *Drosophila*, highlighting its significance in embryonic development [1]. Also, high mobility group box 1 (HMGB1), a mammalian homolog of DSP1, is associated with several neurodegenerative diseases, including Parkinson’s disease (PD), Multiple Sclerosis (MS), and Amyotrophic lateral sclerosis (ALS) [6]. HMGB1-mediated neurodegeneration is often linked to neuroinflammation, a process closely associated with the activation of toll-like receptor 4 (TLR4) and the Receptor for Advanced Glycation End products (RAGEs) [6]. This activation stimulates the production and secretion of pro-inflammatory cytokines, including tumor necrosis factor-alpha (TNF-α) and interleukin-1 (IL-1α and IL-1β) [7]. It has been suggested that HMGB1 plays important roles in both autophagy and apoptosis in neurodegeneration induced by mitochondrial dysfunction. Moreover, HMGB1 contributes to neurodegeneration through multiple pathways, including promoting oxidative stress and disrupting the integrity of the blood–brain barrier (BBB) [8].

**Fig. 1:**
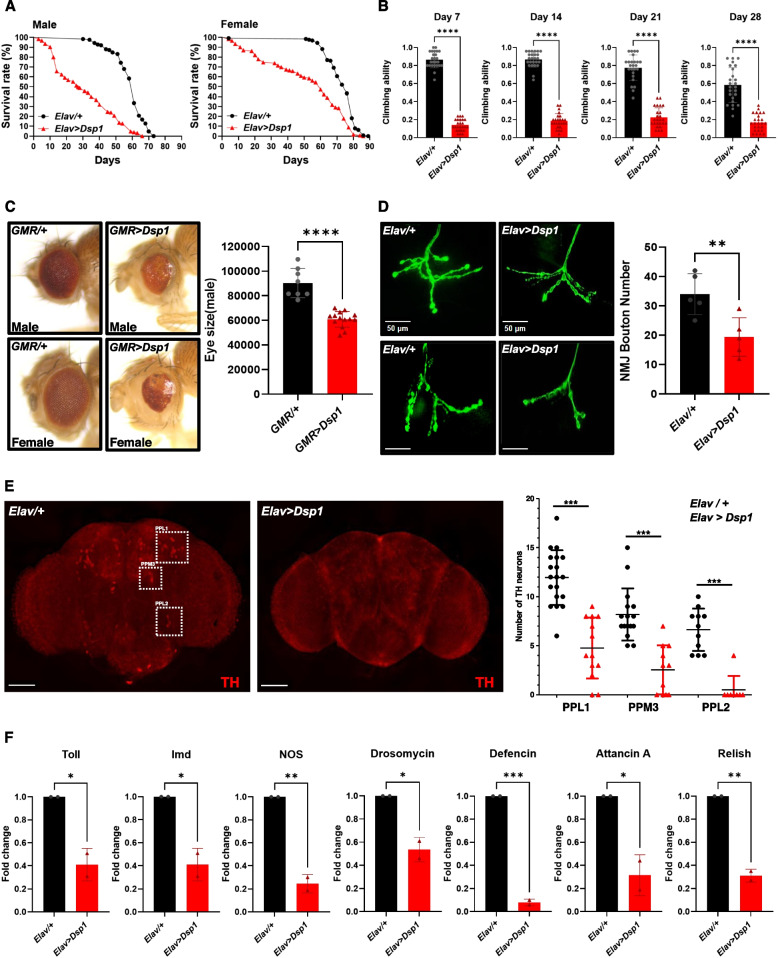
**A** Life span analysis of control and *DSP1*-overexpressed flies*.* The longevity of *DSP1*-overexpressed flies was significantly reduced compare with control for both male and female (N = 125). Data are presented as the mean ± SD. ****p < 0.0001 (Log-rank test). **B** Climbing ability assay of control and *DSP1*-overexpressed flies*.* Neuronal expression of *DSP1*-overexpressed flies significantly reduced climbing ability compared to control in weeks 1–4 (N = 125). Data are presented as the mean ± SD. ****p < 0.0001 (Student’s t-test).** C** Eye phenotype of control and *DSP1*-overexpressed flies using *GMR-GAL4*. Specific *DSP1*-overexpressed flies (*GMR-GAL4* > *UAS-DSP1*) identified a significant reduction in eye size relative to the control (*GMR-GAL4/* +) (N ≥ 8). Data are presented as the mean ± SD. ****p < 0.0001 (Student’s t-test). **D** Fluorescence analysis of third instar larval NMJs under control and *DSP1*-overexpressed flies using anti-horseradish peroxidase (green). Scale bar, 20 µm. *DSP1*-overexpressed flies significantly reduced the number of synaptic boutons relative to control (N = 5). Data are presented as the mean ± SD. **p < 0.01 (Student’s t-test). **E** Fluorescence analysis of the brain in control and *DSP1*-overexpressed flies using anti-tyrosine hydroxylase (red). Scale bar, 20 µm. *DSP1*-overexpressed flies showed significantly decreased DA neurons compared with control. Quantified of dopaminergic neuron number in posterior clusters of the flies’ brain (N ≥ 10). Data are presented as the mean ± SD. ***p < 0.001 (Student’s t-test). **F** RT-PCR for AMPs and Inflammation-related gene expression in control and *DSP1*-overexpressed flies’ brain. *RP49* was used for normalization. *DSP1*-overexpressed flies are significantly reduced AMPs mRNA levels. Data are presented as the mean ± SD. *p < 0.05, **p < 0.01, ***p < 0.001 (Student’s t-test). A,B,D-F Genotypes: control is *Elav-GAL4/* + *(w*.^*1118*^*)*, *DSP1* is *Elav-GAL4/UAS-DSP1*

However, understanding of molecular function of DSP1 in *Drosophila* brain is largely unknown. To address this, we utilized the pan-neuronal specific *Elav-Gal4* system to overexpress DSP1 in neuronal cells, aiming to uncover its functional roles in the brain. Firstly, we measured DSP1 expression levels and the results showed that significant DSP1 upregulation in *DSP1*-overexpressed flies compared to wild-type controls (Supplementary Fig. 1A). Climbing ability and life span serve as indicators of neuronal function in *Drosophila*; a decrease in climbing ability suggests impaired neuronal functions [9]. In accordance with previous studies, we assessed the neuronal function in *DSP1*-overexpressed flies by measuring life span and conducting climbing assays. *DSP1*-overexpressed flies showed a shortened lifespan and defects in climbing ability compared to wild-type flies (Fig. 1 A, B). These data indicate that DSP1 overexpression leads to neuronal impairment in the brain. The *Drosophila* eye has been highlighted as a robust model for assessing toxicity, with eye dysfunction linked to gene expression changes. Indeed, the photoreceptor cells of *Drosophila* have provided a foundation for genetic research into neuronal structure and function. Gene-induced toxicity in the eye is evaluated based on morphological change, proliferation rates, and cell death [9]. Based on this approach, we examined the DSP1-mediated eye morphological changes using *GMR-Gal4*. The overexpression of DSP1 in the eyes showed a reduction of eye size and increased cell death compared with wild-type controls (Fig. 1C). Neuromuscular junction (NMJ) is crucial for signal transduction between motor neurons and muscle fibers, and *Drosophila* NMJ is a well-established model for studying synaptic development, function and plasticity. It serves as a powerful model for studying neurodegeneration [10]. Given this context, we hypothesized that overexpression of DSP1 might lead to abnormalities at the NMJ. Indeed, we observed a decrease in the number of boutons at the NMJ in flies overexpressing DSP1 compared to controls (Fig. 1D), indicating that DSP1 overexpression in neuronal cells could induce motor deficits in *Drosophila*. In motor neurons, dopamine is an essential neurotransmitter involved in regulation of movement and motor control [11]. Tyrosine hydroxylase (TH) is crucial enzyme involved in the biosynthesis of catecholamine, including dopamine. Dysregulation of TH activity can lead to dopamine level abnormalities, affecting behaviors like motor control, learning and memory, and is a pathological hallmark of neurodegenerative diseases [12]. In *DSP1*-overexpressed flies, TH-positive neurons are significantly decreased in PPL1, PPL2, PPM3 clusters (Fig. 1E) and Ple (tyrosine hydroxylase encoding gene) mRNA expression level is also reduced (Supplementary Fig. 1B), known to influence locomotor activity and neuronal functions [13]. Thus, a reduction of TH-positive neurons may contribute to neuronal impairment in DSP1-overexpressed flies. Neurodegeneration can also be driven by excessive inflammatory responses in the brain. *Drosophila* has only innate immune system, demonstrating that dysregulation of this system is a pathological feature in neurodegeneration [14]. Antimicrobial peptides (AMPs), regulated by Toll and Imd signaling pathways, play a role in the innate immune response. We examined that whether DSP1 overexpression manipulates AMPs expression. Upon overexpressing DSP1 in neuron, we found a reduction in AMPs (Drosomycin, Defencin, Attancin A and Relish) and immune-related gene (Toll, Imd and NOS) levels in *DSP1*-overexpressed flies (Fig. 1F). Glia-specific overexpression of DSP1 also downregulated AMPs level (Supplementary Fig. 1C). While an elevated inflammatory response is one of the markers in neurodegeneration, our data suggest that DSP1 overexpression diminishes immune-related gene expression. Previous study has indicated that decreased AMP levels are a critical phenomenon in early neurodegeneration in *Drosophila* [15], suggesting that AMP dysregulation could reflect an immune response imbalance, potentially worsening neuronal damage and disease progression. Therefore, monitoring AMPs levels and their regulation could provide insights into the progression of neurodegenerative diseases. On the other hand, knockdown of DSP1 in neuronal and glial cells showed extended lifespan and improved climbing ability (Supplementary Fig. 1D), we suggest that loss-of-function DSP1 might have neuroprotective effect in *Drosophila*.

In this study, we tried to reveal the functional role of DSP1 in the brain. Our findings indicate that overexpression of DSP1 induces neuronal dysfunction through climbing defect and reduced life span. Furthermore, the overexpression of DSP1 resulted in impaired eye phenotype and decreased TH activity. Based on our observations, we emphasize that DSP1 overexpression strongly linked to neurodegeneration. However, the detailed molecular mechanisms mediated by DSP1 in inducing neurotoxic effects are not fully understood, highlighting the need for further research to unravel the molecular mechanisms of DSP1-mediated neurotoxicity. Given the multifaceted role of HMGB1/DSP1 in neurodegeneration, targeting of HMGB1/DSP1 may represent a novel therapeutic strategy for combating neurodegenerative diseases.

### Supplementary Information

Supplementary Material 1.

Supplementary Material 2.

## Data Availability

All data generated and/or analyzed during this study are included in this published article. Materials and methods are presented in the additional information.

## Abbreviations

DSP1: Dorsal switch protein 1
TBP: TATA-binding protein
TFIIA: Transcription factor IIA
NMJ: Neuromuscular junction
TH: Tyrosine Hydroxylase
HMGB1: High mobility group box 1
PD: Parkinson’ disease
MS: Multiple Sclerosis
ALS: Amyotrophic lateral sclerosis
TLR4: Toll-like receptor 4
RAGEs: Receptor for advanced glycation end products
TNF-α: Tumor necrosis factor-alpha
IL-1: Interleukin-1
BBB: Brain-blood barrier
