# Supplementary material for: The overexpression of DSP1 in neurons induces neuronal dysfunction and neurodegeneration phenotypes in Drosophila
Source: Mol Brain. 2024 Jul 13;17:43. doi: 10.1186/s13041-024-01117-2 (PMC11245852; doi:10.1186/s13041-024-01117-2)
Supplement: Supplementary file 1 — Supplementary Material 1. [file 13041_2024_1117_MOESM1_ESM.docx]

**Supplemental Figure 1**

**A**

**
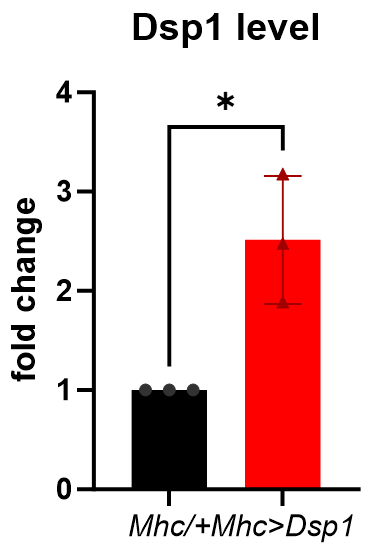
**

**A)** **Overexpression of *Dsp1* in muscle cells increases *Dsp1* gene expression in *Drosophila***. RT-PCR for DSP1 gene expression in control and *Dsp1*-overexpressed flies. *RP49* was used for normalization. *Dsp1*-overexpressed flies are significantly increased *Dsp1* mRNA levels. Data are presented as the mean ± . **p*<0.05 (Student’s t-test). Genotype: control is *Mhc-GAL4/+(w1118)* and Dsp1 is *Mhc-GAL4/UAS-Dsp1*

**B**

**
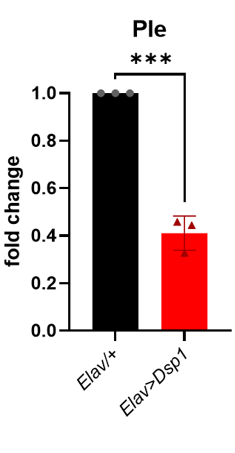
**

**Overexpression of *Dsp1* in neuronal cells reduces *Ple* gene expression in *Drosophila*.** RT-PCR analysis was performed to measure Ple gene expression in the brains of control and *DSP1-*overexpressing flies, with *RP49* used for normalization. The results showed that Ple mRNA levels were significantly reduced in *DSP1*-overexpressing flies. Data are presented as the mean ± SD. ****p*<0.001 (Student’s t-test). Genotype: control is *Elav-GAL4/+(w1118)*, *Dsp1* is *Elav-Gal4/UAS-DSP1*

**C**


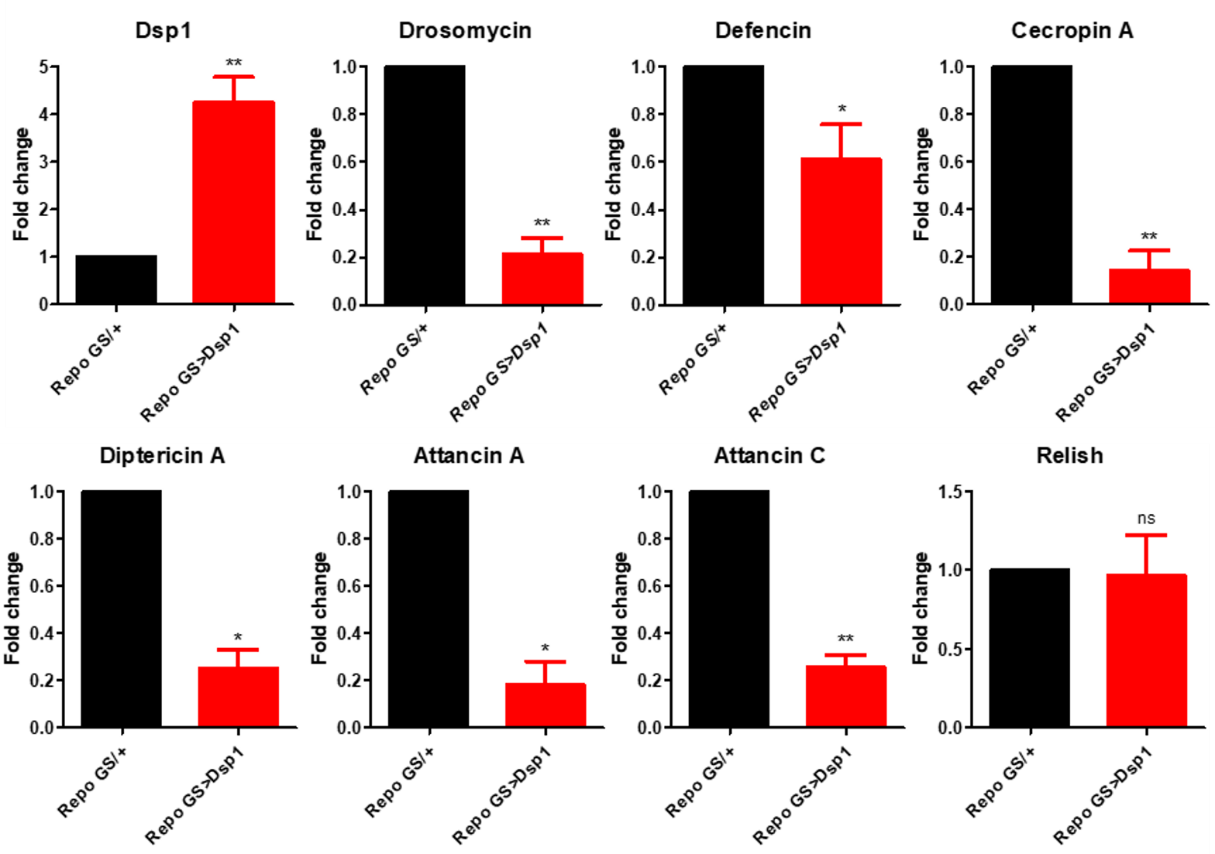


**Overexpression of *Dsp1* in glial cells reduces AMP-related gene expression in *Drosophila*.** RT-PCR analysis was performed to measure AMP gene expression in the brains of control and *DSP1-*overexpressing flies, with *RP49* used for normalization. The results showed that AMP mRNA levels were significantly reduced in *DSP1-*overexpressing flies. Data are presented as the mean ± SD. **p*<0.05, ***p*<0.01, n.s, not significant (Student’s t-test). Genotypes: control is *RepoGS-GAL4/+(w1118)*, *Dsp1* is *RepoGS-GAL4/DSP1*

**D**

**
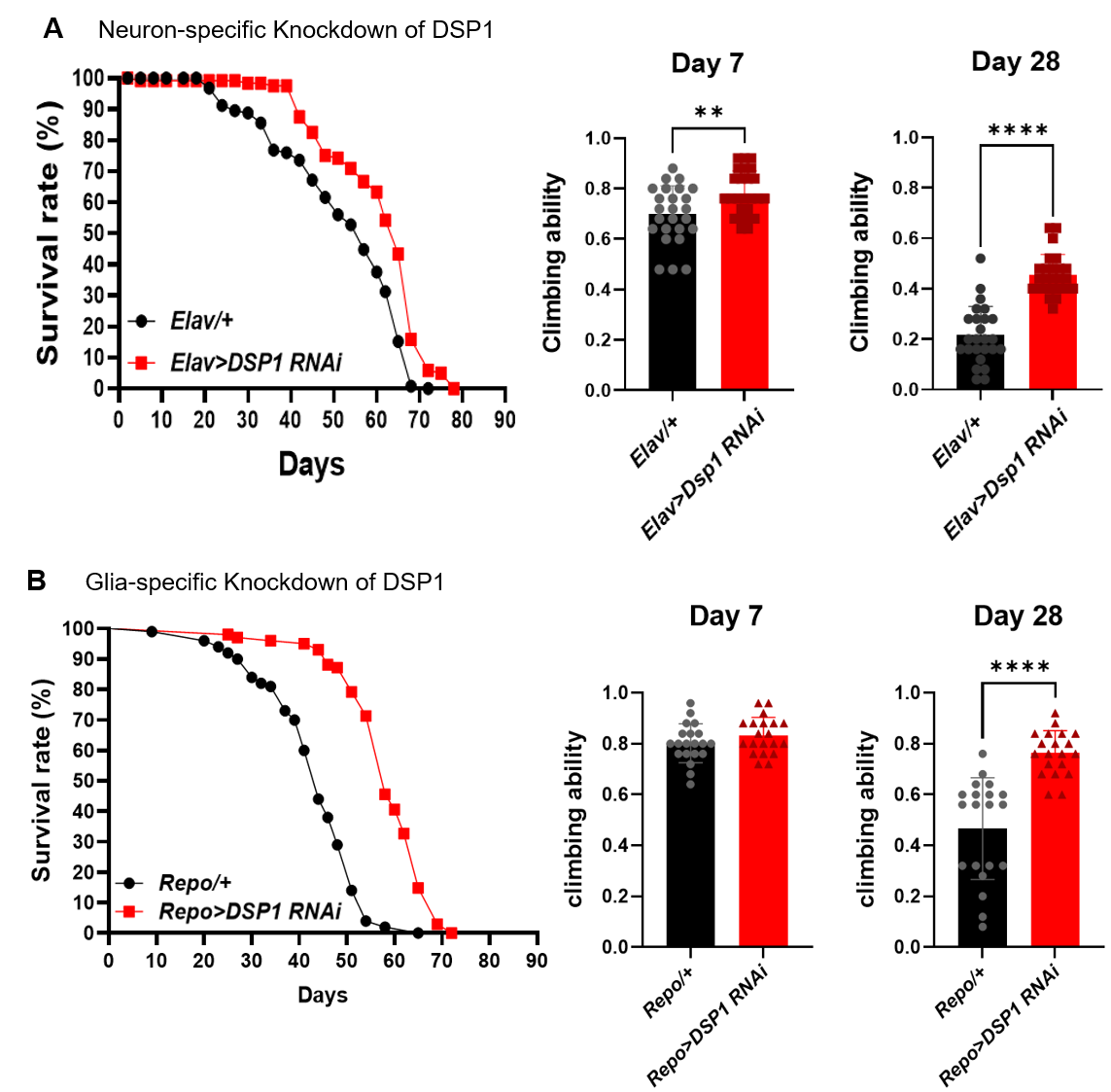
**

**Knockdown of *Dsp1* in neuron and glia induces neuroprotective effect in *Drosophila*.** Lifespan analysis and climbing ability of Control and Dsp1 knockdown flies in neuron (A) and glia (B). Knockdown of Dsp1 was significantly extended lifespan and improved climbing ability compare with control. Data are presented as the mean ± SD. ***p*<0.01, *****p*<0.0001 (Student’s t-test).
