# Supplementary material for: The overexpression of DSP1 in neurons induces neuronal dysfunction and neurodegeneration phenotypes in Drosophila
Source: Mol Brain. 2024 Jul 13;17:43. doi: 10.1186/s13041-024-01117-2 (PMC11245852; doi:10.1186/s13041-024-01117-2)
Supplement: Supplementary file 2 — Supplementary Material 2. [file 13041_2024_1117_MOESM2_ESM.docx]

**Material and Method**

***Drosophila melanogaster* strains**

All stock flies were maintained at 25℃ under normal humidity conditions (60%) and standard yeast medium. Crosses were performed according to the standard procedure, and all progenies were raised at standard condition. *Elav-GAL4*, *GMR-GAL4, RepoGS-GAL4, Repo-GAL4, and UAS-dsp1 RNAi* fly lines were obtained from Bloomington Stock Center (Bloomington, USA). *UAS-dsp1* stocks were obtained from FlyORF (Zurich, Switzerland). We used *W^1118^* flies as controls.

**Climbing assays**

Twenty-five flies were placed in empty column vials and gently tapped to the bottom of the column. After 10 s, flies that climbed above the 2-inch lines on the vial were counted. The rate of flies that passed the target line were recorded. The assay was repeated five times for each vial of flies at 5-minute intervals.

**Lifespan assays**

For longevity measurement, adult male and female flies were collected within 3 days from eclosion. Control and experimental flies were maintained at normal condition at density of 25 same sex flies per vial containing sugar-yeast medium (n = approximately 125). Flies were transferred to fresh food, and dead flies were recorded every 2-3 days.

**Eye measurement**

*GMR-GAL4* flies were crosses with either *W^1118^* or *UAS-dsp1* flies, and the progenies were scored for *DSP1* effects in the eyes. Eye images were captured using a IMT cam, and eye size was measured using Image J v1.44 software (National Institute of Health, Bethesda, USA).

**Immunofluorescence staining**

For larvae NMJ staining, third instar larvae were dissected in PBS, fixed in 4% paraformaldehyde for 20 min, and washed three times in 0.1% Triton-100 in PBS(PBST). Tissues were blocked with 4% BSA in PBST for 30min at room temperature. FITC-conjugated anti-HRP (Jackson Immunoresearch, USA) was used at 1:100 and incubated at 4℃ overnight. After washing, tissues were mounted in VECTASHIELD Antifade mounting medium with DAPI (VECTOR laboratories, USA). Boutons were stained with an anti-HRP antibody. The boutons were recorded if they were round. For staining DA neurons in fly brain, adult flies head were dissected in PBS. Fixation and blocking were carried out through the same process as before. The Brains were incubated overnight at 4℃ in Primary antibody, anti-Tyrosine Hydroxylase (EMD Millipore, Germany), diluted at 1:300. The primary antibody was removed by washing with PBT and secondary antibody, Goat anti-rabbit IgG, FSD^TM^ 594 (Bioacts, Korea), diluted at 1:500 and added. An incubation was 2h long at the room temperature. The antibody was removed by washing in PBST and mounted in same solution before. The number of DA neurons was counted by dividing into three cluster area in flies’ posterior brain. All immunofluorescence images were captured using a camera stereo microscope (Olympus SZ61, Japan).

**Quantitative RT-PCR**

RNA was extracted from *Drosophila* adult head (*ELAV-GAL4*) and thorax (*Mhc-GAL4*) with easy-BLUE (INtRON Biotechnology, Korea) and reverse transcribed by using High-Capacity cDNA Reverse Transcription Kit (Applied Biosystems, USA). Quantitative PCR was performed by using Power SYBR™ Green PCR Master Mix (Applied Biosystems™, USA). Each mRNA level was normalized by *RP49* mRNA levels. Primers used were as follows: Drosomycin (F:TACTTGTTCGCCCTCTTCG and R: GTATCTTCCGGACAGGCAGT), Defensin ( F:GTTCTTCGTTCTCGTGG and R: CTTTGAACCCCTTGGC), Attancin A (F: CAATGGCAGACACAATCTGG and R: ATTCCTGGGAAGTTGCTGTG), Relish (F: GGCATCATACACACCGCCAAGAAG and R: GTAGCTGTTTGTGGGACAACTCGC), Toll (F: TGTGCTCAGCCAGAACTTTATCA and R: CGCCGCCCTGAATTCA), Imd (F: GCGACGCGCAAAACG and R: ACAGCGCCCAGCAACAG), NOS (F: GCATTTCGAGAACGAGTCCAA and R: GCACGATCCAAATCCAATCAG), Ple (F: TGAGGAGGATGTTGAGTTTG and R: GCACTTAAATCCAATGTCGCA), Dsp1 (F: GCCCCATGAGCAGAGTCAAG and R: AAGTATGCGTAGGCGGTCATTC) and RP49 (F: AGGGTATCGACAGAGTG and R: CACCAGGAACTTCTTGAATC).

**Statistical analysis**

All statistical analysis was performed with Prism (Graphpad, USA) version 10.2.0 and *p* values were calculated with Student’s t-tests and log-rank tests. Differences were considered significant when **p*<0.05; ***p*<0.01; ****p*<0.001; *****p*<0.0001.
